# Is It Professional?

**Published:** 1880-06

**Authors:** 


### SPECIAL COMMUNICATIONS.

---

### IS IT PROFESSIONAL?

MR. EDITOR: I desire to ask if the conduct of a physician of this city, as I relate it, is professional, that I may be able to judge rightly of such conduct, should I again find myself treated in a like manner. I was called by parents to see a sick child; for all I know I gave satisfaction; the child was better after a day or two; my bill was paid, and the understanding was, if the child became worse, I was to be sent for. The same evening symptoms which I had spoken of to the parents, appeared, and I was hastily called, but was out of my office; the parents alarmed sent for another physician. For some reason, unknown to me, the last physician is retained; whether by the express wish of the parents only, or whether they were influenced in any way by the new comer, I know not. I met the father a few days after on the street, and he told me what I have related, casually adding that the principal remedy I had given was continued, while the conversation showed that some symptoms, which I had recognized, had been impressed upon him, as vastly more important than I had thought them, or expressed them to be.

In the code of ethics of the Massachusetts Medical Society, adopted Feb. 4, 1880, appear the following "principles and rules of action for their (members) guidance and convenience." Rule II says: "The kind of competition which might be considered honorable in business cannot exist among physicians without diminishing their usefulness and lowering the standard of the medical profession." Rule IV says: "in his relation with another practitioner and his patient, a physician should be governed by strict rules of honor and courtesy." Rule IV, § 1-2-3, says: "§ 1. A physician should take no steps with a view directly or indirectly to divert to himself the patient or practice of another physician.

"§ 2. If formally requested to take charge of a patient or family, usually attended by another physician, he should consent to do so only after notifying the latter—unless the case be one of pressing necessity.

"§ 3. If a physician is called during the temporary absence * * of another physician, * * he should direct the former be sent for as soon as he is able to take charge of the case."

I know we are not governed by the *rules* of the Massachusetts society, but are we not by the same *principles* of "strict honor and courtesy?"

I am a young man, and the loss of this patient to me is an important matter; the physician who succeeded me has been in practice many years, and, to him, I suppose the gain of my patient was a small matter; but, we accidently met the next morning, as he was going into the house of *our* patient, and he passed me without notice. If the principles here given are not recognized among Buffalo physicians; if ordinary business competition is the rule; if selfishness, and all the underhanded methods of getting one another's patients, so well known to all, or, so easily learned, are the guiding principles among us, I want to know it early in my career, so that I may meet trickery by being forewarned, and be able to do what I can, as I grow to practice, and perhaps to influence, to instill a higher principle of "honor and courtesy" among us. But, if these are the rules, which I very much doubt, there are, as I have found out in my short experience in Buffalo, many who fail to keep them, for, in several somewhat similar cases, I have been treated with singular "honor and courtesy" by some of our oldest and most influential physicians. I write sincerely, for I am truly ignorant, when I ask the question "Is it professional?" Can you tell me, Mr. Editor?

A Young Physician.

